# Non-Invasive Detection of Fibrotic NASH in NAFLD Patients with Low or Intermediate FIB-4

**DOI:** 10.3390/jcm11154394

**Published:** 2022-07-28

**Authors:** Katharina John, Martin Franck, Sherin Al Aoua, Monika Rau, Yvonne Huber, Joern M. Schattenberg, Andreas Geier, Matthias J. Bahr, Heiner Wedemeyer, Klaus Schulze-Osthoff, Heike Bantel

**Affiliations:** 1 Department of Gastroenterology, Hepatology and Endocrinology, Hannover Medical School, Carl-Neuberg-Strasse 1, 30625 Hannover, Germany; john.katharina@mh-hannover.de (K.J.); franck.martin@mh-hannover.de (M.F.); alaoua.sherin@mh-hannover.de (S.A.A.); wedemeyer.heiner@mh-hannover.de (H.W.); 2 Division of Hepatology, Department of Internal Medicine II, University Hospital Würzburg, 97080 Würzburg, Germany; rau_m@ukw.de (M.R.); geier_a2@medizin.uni-wuerzburg.de (A.G.); 3 Department of Internal Medicine I, University Medical Center Mainz, 55131 Mainz, Germany; yvonne.huber@unimedizin-mainz.de (Y.H.); joern.schattenberg@unimedizin-mainz.de (J.M.S.); 4 Brandenburg Medical School, University Hospital Ruppin-Brandenburg, 16816 Neuruppin, Germany; matthias.bahr@gmx.de; 5 Interfaculty Institute of Biochemistry, University of Tübingen, 72076 Tübingen, Germany; kso@uni-tuebingen.de; 6 German Cancer Consortium (DKTK), German Cancer Research Centre (DKFZ), 69120 Heidelberg, Germany

**Keywords:** apoptosis, biomarker, fibrosis, FIB-4, NAFLD, NASH, keratin-18, M30

## Abstract

Background: Non-alcoholic steatohepatitis (NASH) and fibrosis are the main prognostic factors in non-alcoholic fatty liver disease (NAFLD). The FIB-4 score has been suggested as an initial test for the exclusion of progressed fibrosis. However, increasing evidence suggests that also NASH patients with earlier fibrosis stages are at risk of disease progression, emphasizing the need for improved non-invasive risk stratification. Methods: We evaluated whether the apoptosis biomarker M30 can identify patients with fibrotic NASH despite low or intermediate FIB-4 values. Serum M30 levels were assessed by ELISA, and FIB-4 was calculated in an exploration (*n* = 103) and validation (*n* = 100) cohort of patients with histologically confirmed NAFLD. Results: The majority of patients with low FIB-4 (cut-off value < 1.3) in the exploration cohort revealed increased M30 levels (>200 U/L) and more than 80% of them had NASH, mostly with fibrosis. NASH was also detected in all patients with intermediate FIB-4 (1.3 to 2.67) and elevated M30, from which ~80% showed fibrosis. Importantly, in the absence of elevated M30, most patients with FIB-4 < 1.3 and NASH showed also no fibrosis. Similar results were obtained in the validation cohort. Conclusions: The combination of FIB-4 with M30 enables a more reliable identification of patients at risk for progressed NAFLD and might, therefore, improve patient stratification.

## 1. Introduction

Non-alcoholic fatty liver disease (NAFLD) represents one of the most common etiologies of chronic liver diseases, ranging from non-alcoholic fatty liver (NAFL) to non-alcoholic steatohepatitis (NASH). NASH is characterized by the presence of liver steatosis together with lobular inflammation and hepatocellular ballooning as histological signs of liver injury. Patients with NASH have an increased risk of liver disease progression with development of liver fibrosis/cirrhosis. Of note, liver fibrosis represents the main risk factor not only for liver-related but also for overall mortality [1,2]. Liver biopsy remains the gold standard for NASH detection and fibrosis staging. However, with respect to the NAFLD prevalence of 25%, it is unrealistic to perform liver biopsies in the whole population [3]. Non-invasive scoring systems are, therefore, increasingly used to identify NAFLD patients at risk of advanced fibrosis and hence clinical complications.

The FIB-4 score is based on simple parameters including age, aminotransferases, and platelet count and is, therefore, easy to perform [4]. This test considers a low (<1.3) and a high (>2.67) cut-off value to rule out or rule in advanced fibrosis [4]. Because of its high negative predictive value (>90%) for the exclusion of advanced fibrosis, it is recommended as an initial approach to exclude progressed NAFLD in clinical practice [5]. However, there is increasing evidence that also NASH patients with earlier fibrosis stages are at risk for disease progression and complications [1,2]. Furthermore, about one-third of patients fall between the upper and lower cut-off value, giving an indeterminate result for fibrosis assessment by FIB-4 [4,6]. Therefore, the combination of FIB-4 with a non-invasive test that allows an earlier identification of NAFLD patients at risk for disease progression might improve non-invasive patient stratification.

Hepatocyte apoptosis plays an important role in NAFLD progression and fibrosis development [7,8]. During apoptosis, activated caspases cleave the intermediate filament protein keratin-18 (K18) that is expressed by hepatocytes [9,10]. Caspase-cleaved K18 fragments are released from apoptotic hepatocytes and can be detected by the M30 enzyme-linked immunosorbent assay (ELISA) [11]. This ELISA has been extensively evaluated in various NAFLD studies demonstrating that caspase-cleaved K18 fragments (M30 levels) are significantly increased in sera from NASH compared to NAFL patients [12,13,14,15]. Studies with paired biopsies further showed that histological changes in the NAFLD activity score (NAS) significantly correlated with M30 levels in the blood of NAFLD patients [16,17]. Moreover, a recent study demonstrated a robust dose-dependent reduction in M30 levels in NASH patients receiving drug therapy compared to placebo treatment [18]. M30 might, therefore, represent a sensitive diagnostic biomarker for the non-invasive assessment of NAFLD activity.

In this biopsy-proven multicenter study, we evaluated whether detection of M30 levels in NAFLD patients with excluded advanced or indeterminate fibrosis by FIB-4 score (<1.3 or 1.3–2.67) might improve risk stratification and could be helpful for decision making regarding which patients should be referred to secondary care centers and considered for liver biopsy.

## 2. Methods

### 2.1. Patients

We investigated 103 patients (exploration group) with biopsy-proven NAFLD from Hannover Medical School. A total of 76 of those patients revealed NASH with the simultaneous presence of liver steatosis, lobular inflammation, and hepatocellular ballooning [19,20]. Histological fibrosis was assessed according to Kleiner et al. [21] and could be detected in 50% of the NAFLD patients, 38% of whom revealed fibrosis stages ≥ F2. Patients with other co-existing liver diseases were excluded.

In addition, we investigated further 100 biopsy-proven NAFLD patients from the University Hospitals of Würzburg and Mainz (validation cohort). NASH was detected in 65 patients of this cohort and 66% of the NAFLD patients revealed fibrosis, 50% of whom with ≥ F2.

Clinical and histological characteristics of both patient cohorts have been previously described [22]. The mean age was 45.8 ± 12.3 years in the exploration cohort and 47.7 ± 13.1 years in the validation cohort. Mean levels for AST and ALT were 54.0 ± 29.8 U/L and 90.3 ± 56.2 U/L in the exploration and 50.2 ± 35.0 U/L and 72.8 ± 64.5 U/L in the validation cohort. Mean platelet count was 237.5 ± 58.5 Tsd/µL and 252.0 ± 85.5 Tsd/µL in the exploration and validation cohort. A total of 16.5% of NAFLD patients in the exploration and 38.0% of patients in the validation cohort revealed type 2 diabetes. The study was performed according to the guidelines of the local ethics committees.

### 2.2. Determination of FIB-4 Score

For FIB-4, which is based on the combination of age, AST, ALT, and platelet count, two cut-offs to rule out (<1.3) or to rule in (>2.67) advanced fibrosis in NAFLD are used [4]. According to those cut-offs, patients are divided into low risk (<1.3), intermediate risk (1.3–2.67), or high risk (>2.67) for advanced fibrosis. This score has been recommended in guidelines for the initial risk stratification of NAFLD patients and for decision making regarding which patient should be referred to secondary care centers and considered for liver biopsy [5].

### 2.3. Serological Detection of M30 Levels

For the measurement of caspase-cleaved K18 in sera from NAFLD patients, we used the M30-Apoptosense ELISA (PEVIVA/VLVbio, Nacka, Sweden) as described [11,13]. All samples were analyzed in duplicates.

### 2.4. Statistical Analysis

Univariate logistic regression analysis was performed in patients with low or intermediate FIB-4 (≤2.67) to identify variables possibly associated with NASH and simultaneous fibrosis ≥ F2 (age, sex, AST, ALT, diabetes mellitus, BMI, and M30). Variables associated with NASH and fibrosis ≥ F2 in the univariate analysis (*p* < 0.1) were further considered for multivariate regression analysis. The odds ratio for M30 to predict NASH with fibrosis ≥ F2 in NAFLD patients with low or intermediate FIB-4 (≤2.67) was calculated by using SPSS 27 software (IBM Corporation, Armonk, NY, USA). A *p* value of less than 0.05 was considered significant.

## 3. Results

### 3.1. Serological Detection of Caspase-Cleaved Keratin-18 (M30) Enables the Identification of NAFLD Patients with Fibrotic NASH despite Low FIB-4

The FIB-4 index is currently recommended as an initial test for risk stratification of NAFLD patients. This non-invasive test is easy to perform and allows the exclusion of advanced fibrosis with a high negative predictive value (NPV > 90%) [5]. NAFLD patients with excluded advanced fibrosis by FIB-4 (cut-off < 1.3) usually remain in primary care for disease monitoring and are not referred to secondary care for further risk stratification. However, there is increasing evidence that also NASH patients with early fibrosis stages are at risk for disease progression and developing of hepatic and extrahepatic complications [1,2,23]. Since apoptosis plays an early role for disease progression and fibrosis development in NAFLD, we asked whether the serological detection of caspase-cleaved keratin-18 fragments (M30) could identify NAFLD patients with fibrotic NASH despite low FIB-4 values (<1.3). We used the M30 cut-off >200 U/L which has been previously shown to detect progressed NAFLD with appropriate diagnostic performance [12,22].

**Figure 1:**
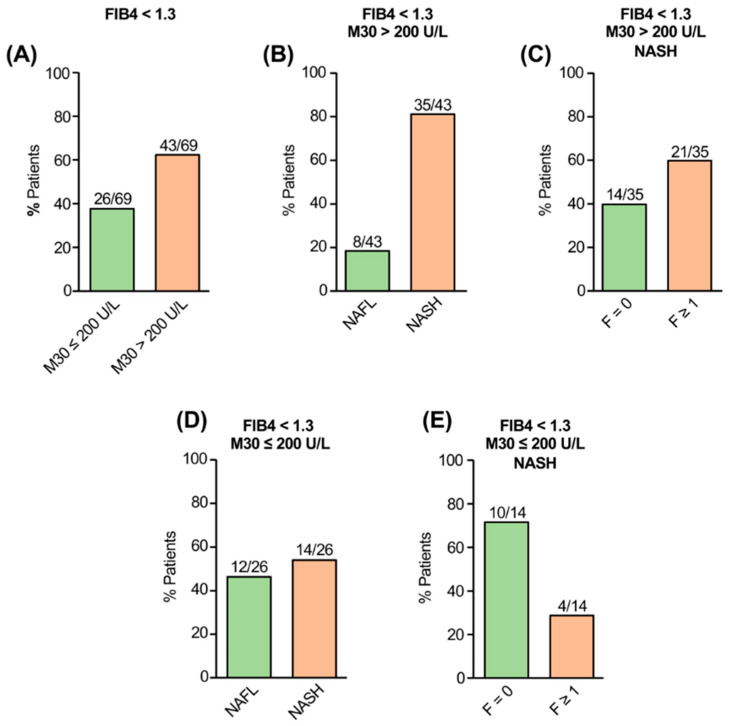
Serological detection of caspase-cleaved K18 by the M30 ELISA in NAFLD patients with low FIB-4 (exploration cohort). Most patients with FIB-4 < 1.3 showed elevated (>200 U/L) M30 levels (**A**). Most patients with FIB-4 < 1.3 and elevated M30 levels revealed NASH (**B**). Patients with NASH and elevated M30 levels despite low FIB-4 revealed liver fibrosis in the majority of cases (**C**). Vice versa, NAFLD patients with FIB-4 < 1.3 and non-elevated M30 (≤200 U/L) showed NAFL or NASH without fibrosis in the majority of cases (**D**,**E**).

In the exploration cohort (*n* = 103), an absence of advanced fibrosis was indicated by FIB-4 < 1.3 in 69 patients. The majority (43/69) of NAFLD patients with low FIB-4, however, showed elevated M30 levels (>200 U/L) and 81% of them revealed NASH (Figure 1A,B). In 60% (21/35) of those patients, fibrosis was histologically detected (F1 = 12; F2 = 7; F3 = 2; Figure 1C). Vice versa, from patients with FIB-4 < 1.3 and M30 ≤ 200 U/L, 46% (12/26) showed NAFL whereas 14 patients had NASH (Figure 1D), in the majority of cases without fibrosis (F0 = 10; F1 = 3, F2 = 1; Figure 1E).

**Figure 2:**
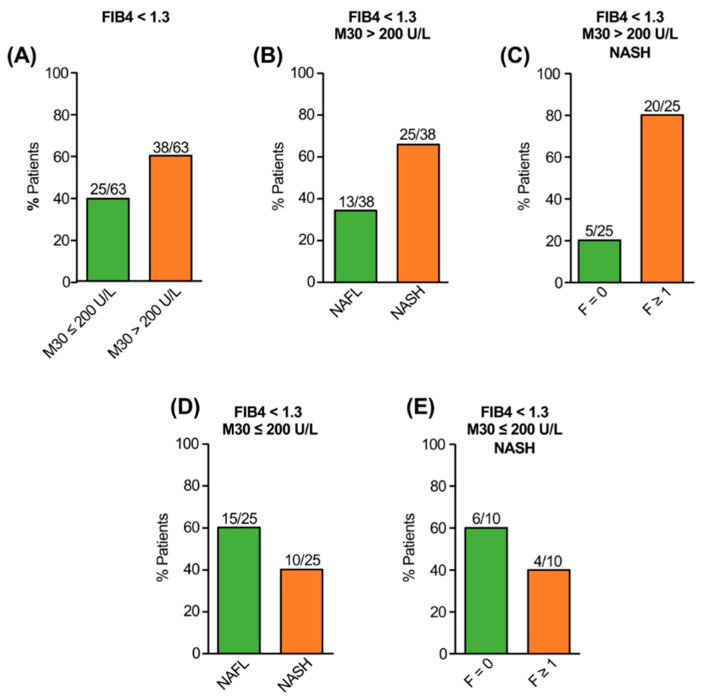
Validation of the M30 marker for the detection of NASH and fibrosis in NAFLD patients with low FIB-4. In the majority of NAFLD patients with FIB-4 < 1.3 elevated M30 levels (>200 U/L) could be detected (**A**), and most of them revealed NASH (**B**). Most patients with NASH and elevated M30 showed histological fibrosis despite FIB-4 < 1.3 (**C**). Vice versa, most NAFLD patients with FIB-4 < 1.3 and non-elevated M30 levels revealed NAFL (**D**). Most patients with NASH despite low FIB-4 and non-elevated M30 levels did not show histological fibrosis (**E**).

In the validation cohort (*n* = 100), 63 patients revealed FIB-4 values < 1.3 and, in accordance with the exploration cohort, the majority (38/63) of them had M30 levels > 200 U/L (Figure 2A). Patients with elevated M30 despite low FIB-4 had NASH in 66% of cases (25/38; Figure 2B), and most of them histologically showed fibrosis (F1 = 12; F2 = 6; F3 = 2; Figure 2C). Vice versa, patients with low FIB-4 and non-elevated M30 had NAFL in the majority of cases (60%; Figure 2D), and those patients who revealed NASH despite non-elevated M30 had mostly no or only minimal fibrosis (F0 = 6, F1 = 3, F2 = 1; Figure 2E). These data indicate that NAFLD patients with low FIB-4 but elevated M30 levels revealed fibrotic NASH to a significant proportion (51%; 41/81), whereas patients with low M30 mostly showed NAFL. Moreover, patients with NASH despite low FIB-4 and non-elevated M30 had no histological evidence of advanced fibrosis.

### 3.2. M30 Improves Risk Stratification of NAFLD Patients with Intermediate FIB-4

NAFLD patients with intermediate (1.3–2.67) FIB-4 are usually referred to secondary care centers to further rule out/in advanced fibrosis, e.g., by transient elastography and/or liver biopsy. We, therefore, asked whether the detection of M30 could improve risk stratification in patients with intermediate FIB-4 and, thus, reduce the referral rate in this situation.

**Figure 3:**
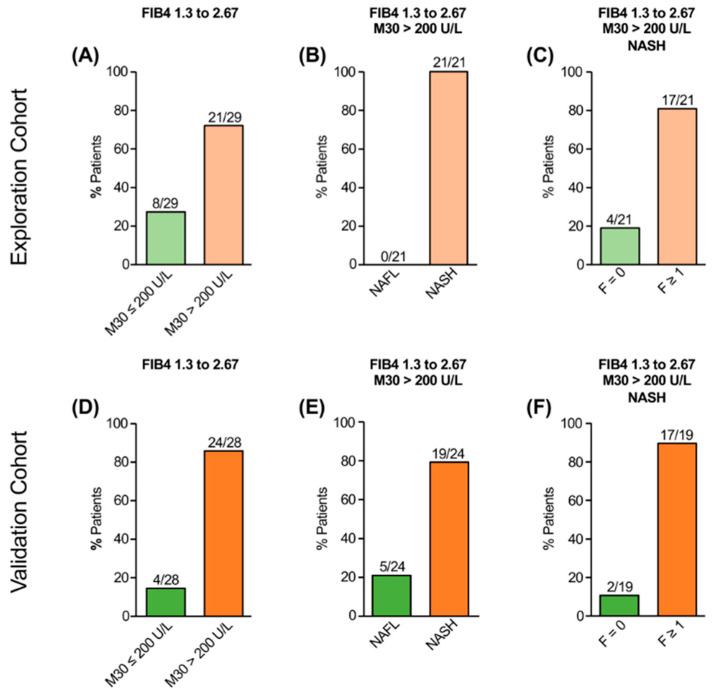
Serological detection of M30 levels in NAFLD patients with intermediate FIB-4 (1.3–2.67). In the exploration cohort, NAFLD patients with intermediate FIB-4 had elevated M30 levels in the majority of cases (**A**). All patients with elevated M30 levels showed NASH (**B**) and the majority of them revealed fibrosis (**C**). Most NAFLD patients with intermediate FIB-4 in the validation cohort showed elevated M30 levels (**D**) and the majority of them revealed NASH (**E**), mostly with fibrosis (**F**).

In the exploration cohort, 28% (29/103) of NAFLD patients revealed intermediate FIB-4 values (Figure 3A), and the majority of them (21/29) had elevated M30 levels (>200 U/L). All patients with intermediate FIB-4 and elevated M30 levels had NASH (Figure 3B), most of them (81%) with fibrosis (F1 = 9, F2 = 2, F3 = 3, F4 = 3; Figure 3C). Among the patients with intermediate FIB-4 and non-elevated M30 (≤ 200 U/L, *n* = 8), six patients had NAFL and two had NASH without fibrosis. A similar number of NAFLD patients in the validation cohort (28%) had intermediate FIB-4 values, 86% (24/28) of them with elevated M30 levels (Figure 3D). A total of 79% (19/24) of those patients showed NASH (Figure 3E). In the majority of cases (89%), fibrosis was histologically detected (F1 = 4, F2 = 8, F3 = 4, F4 = 1, Figure 3F). From the four patients without elevated M30, two patients had NAFL and two patients had NASH without fibrosis or with non-advanced fibrosis, respectively. Among the total number of NAFLD patients with intermediate FIB-4 and non-elevated M30 (*n* = 12), almost all patients (*n* = 11) revealed no histological evidence for NASH with significant fibrosis.

### 3.3. Diagnostic Performance of M30 to Predict NASH with Significant Fibrosis in NAFLD Patients with Low or Intermediate FIB-4

Having demonstrated that M30 is suitable for the detection of fibrotic NASH in NAFLD patients with low or intermediate FIB-4, we evaluated its diagnostic performance for ruling out or in the diagnosis NASH with significant fibrosis (≥F2). These patients are especially at risk for disease progression and the development of liver-related complications, and are, therefore, considered for the inclusion in clinical trials evaluating novel NASH therapeutics.

**Figure 4:**
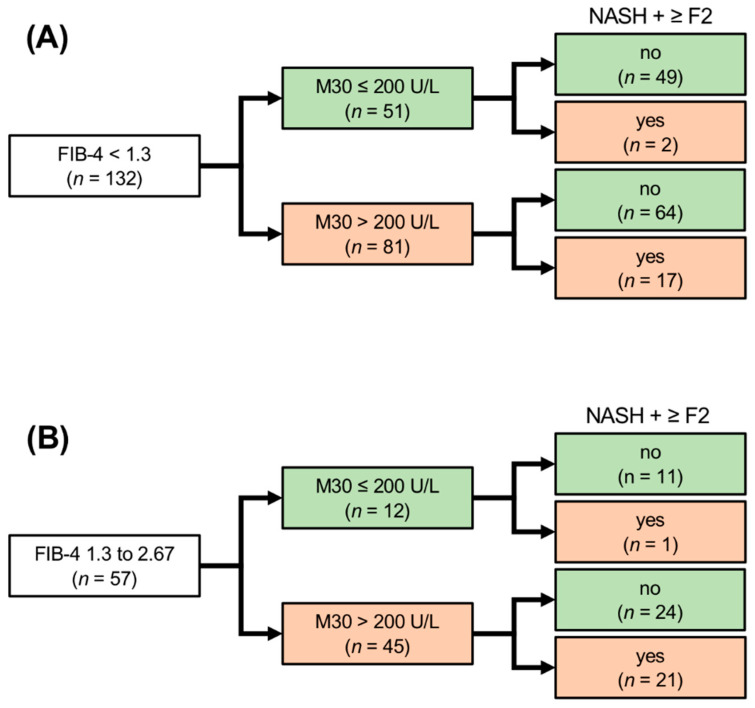
Detection of NASH and significant fibrosis (≥F2) in NAFLD patients with low (<1.3) or intermediate (1.3–2.67) FIB-4 by M30. NAFLD patients of the exploration and validation cohort with low FIB-4 showed elevated M30 levels in the majority of cases and a considerable proportion of them (21%) revealed NASH with significant fibrosis (**A**). Vice versa, almost all patients with low FIB-4 and non-elevated M30 in the overall cohort did not show NASH with fibrosis ≥ F2 (**A**). Most NAFLD patients of the exploration and validation cohort with intermediate FIB-4 showed elevated M30 levels and almost half of them had NASH with significant fibrosis (**B**). Most NAFLD patients with intermediate FIB-4 and non-elevated M30 did not show NASH with significant fibrosis (**B**).

NAFLD patients with low FIB-4 (<1.3) in the overall cohort (*n* = 132) showed non-elevated M30 levels in 39% of cases (51/132), from which 96% (49/51) did not fulfill the criterion NASH with significant fibrosis (≥F2). The NPV and sensitivity of M30 for the exclusion of NASH with significant fibrosis in patients with low FIB-4 were 96% and 89%, respectively. A total of 61% (81/132) of patients with low FIB-4 revealed elevated M30, and 21% of them (17/81) had NASH with significant fibrosis, resulting in a specificity of 43% and a positive predictive value (PPV) of 21% (Figure 4A).

NAFLD patients with intermediate FIB-4 (1.3–2.67) in the overall cohort (*n* = 57), which are usually referred to secondary care centers for further risk stratification, showed non-elevated M30 levels in 21% of cases (12/57). Almost all of them (11/12) did not fulfill the criterion NASH with significant fibrosis. The NPV and sensitivity of M30 for the exclusion of NASH with significant fibrosis in NAFLD patients with intermediate FIB-4 were 92% and 95%. NAFLD patients with intermediate FIB-4 showed elevated M30 in 79% of cases (45/57) and almost half of them (47%) revealed NASH with fibrosis ≥ F2, resulting in a specificity of 31% and a PPV of 47% (Figure 4B).

In NAFLD patients with low or intermediate FIB-4 (≤2.67, *n* = 189), the multivariate regression analysis revealed that elevated M30 levels represent an independent risk factor for having NASH with significant fibrosis with an odds ratio of 5.4 (95% CI: 1.5–19.4; *p* = 0.01).

According to the current recommendations, patients with low FIB-4 remain in primary care without additional risk evaluation, whereas patients with intermediate or high FIB-4 are considered for further risk stratification and, therefore, are referred to secondary care [5]. When we considered patients with high FIB-4 (>2.67) and patients with FIB-4 ≤ 2.67 who revealed elevated M30 levels for further risk stratification, the overall referral rate could be reduced by 31% (63/203). Using this algorithm, the NPV and sensitivity for the exclusion of NASH with significant fibrosis was improved by 9% and 32%, respectively, compared to the use of FIB-4 score alone. When patients with low FIB-4 (<1.3) were not considered for additional risk evaluation, and only patients with either high FIB-4 or with intermediate FIB-4 and elevated M30 levels were considered for further risk stratification, the overall referral rate could even be reduced by 71% (144/203). In this setting, the specificity and PPV to detect NASH with significant fibrosis were each improved by 7%, compared to the use of the FIB-4 score alone.

## 4. Discussion

NAFLD affects ~25% of the global population and has become the most common chronic liver disease seen in primary care, ranging from NAFL to NASH with the risk of developing advanced liver fibrosis [3,24,25]. The diagnosis of NASH is important since it is associated with faster liver fibrosis progression [5,26]. Hepatic fibrosis represents a major risk factor for developing hepatic as well as extrahepatic complications and mortality [1,27]. The identification of NAFLD patients at risk for disease progression, i.e., patients with fibrotic NASH, is, therefore, of clinical relevance. Liver biopsy currently represents the reference standard for the diagnosis of NASH and fibrosis staging. However, this invasive procedure is costly and remains associated with clinical complications and sampling error [28,29]. Furthermore, it is logistically challenging and unrealistic to perform a liver biopsy in 25% of the general population. Therefore, the non-invasive risk stratification of NAFLD patients represents an urgent clinical need.

FIB-4 is recommended as the first step for ruling out advanced fibrosis in NAFLD [5]. However, previous studies found an insufficient sensitivity (69–74%) for FIB-4 < 1.3 [4,30,31] with false-negative results of 29% [30]. To further improve the first-line procedure for ruling out advanced fibrosis in NAFLD, the combination with an additional non-invasive test has been suggested [30]. Indeed, the sequential combination of FIB-4 and transient elastography (TE) could reduce the false-negative rate for advanced fibrosis [32]. Since TE is commonly not available in primary care centers, the combination of FIB-4 with a blood-based test might represent a more practicable method to improve the diagnostic performance and primary risk stratification in NAFLD.

The M30 marker has been demonstrated to correlate with histological features of NASH, i.e., lobular inflammation and hepatocellular ballooning, which are both related to NAFLD progression and fibrosis development [12,25,33,34,35]. Moreover, there is evidence that M30 levels increase with fibrosis development and progression [12,13,34,36]. Inflammation triggers monocyte recruitment and hepatocellular apoptosis which results in stellate cell activation and fibrogenesis [37,38]. In the present study, we, therefore, evaluated whether the combination of FIB-4 with M30 could be beneficial for the initial risk stratification of NAFLD patients.

In our overall cohort, about half of the NAFLD patients with low FIB-4 and elevated M30 showed histologically fibrotic NASH, and ~40% of them had already developed significant fibrosis (≥F2). Patients with low FIB-4 and non-elevated M30 revealed NAFL or NASH with no or minimal fibrosis in the majority of cases. For NAFLD patients with low FIB-4 (*n* = 132), no further risk stratification is currently recommended. However, the additional use of M30 in those patients reduced the rate of false-negative results from 37% (49/132) to 6% (8/132) for NASH with fibrosis ≥ F1 and from 14% (19/132) to 1.5% (2/132) for NASH with fibrosis ≥ F2. These data indicate that patients with low FIB-4 and elevated M30 are at risk for fibrotic NASH and faster disease progression. These patients might, therefore, benefit from further risk stratification, e.g., by TE.

For NAFLD patients with intermediate FIB-4, an additional non-invasive test is recommended to reduce the referral rate and need for liver biopsy [5,39,40]. It could be demonstrated that the enhanced liver fibrosis (ELF) score, which considers various markers of extracellular matrix remodeling and fibrogenesis, improves the detection of advanced fibrosis and reduces the referral rate in NAFLD patients with intermediate FIB-4 [40]. However, measurement of the ELF score is complex, costly, and not broadly available. In our study, we evaluated M30 levels in patients with intermediate FIB-4, which is easy to apply and already used in clinical NASH trials [18]. We could demonstrate that 76% (34/45) of NAFLD patients with intermediate FIB-4 and elevated M30 showed fibrotic NASH and 47% (21/45) of those patients revealed already significant fibrosis. Moreover, when considering only patients with high FIB-4 (>2.67) and patients with intermediate FIB-4 who revealed elevated M30, the overall referral rate to secondary care could be reduced by 71%. Patients with intermediate FIB-4 might, therefore, benefit from M30 detection for further risk stratification.

A limitation of our study could be that the NAFLD patients were recruited at tertiary centers. Thus, progressed NAFLD may be overrepresented in comparison to the general population. However, the M30 revealed a similar diagnostic performance for the identification of patients with fibrotic NASH in the exploration and validation cohort, although the proportion of patients with diabetes and, therefore, the risk of progressed disease was higher in the validation cohort.

In conclusion, elevated M30 levels could identify NASH with significant fibrosis in a remarkable proportion of NAFLD patients even with low FIB-4. In NAFLD patients with intermediate FIB-4, the additional use of the M30 marker could considerably reduce the referral rate. The detection of M30 in NAFLD patients with low or intermediate FIB-4 might, therefore, improve risk stratification and contribute to decision making regarding which patient should be referred to secondary care and considered for liver biopsy. Further large-cohort studies are required to evaluate the suitability of M30 in combination with FIB-4 for the risk stratification of NAFLD patients.

## Data Availability

The data presented in this study are available on reasonable request from the corresponding author.
